# Participants' perceptions of a lifestyle approach to promoting physical activity: targeting deprived communities in Kingston-Upon-Hull

**DOI:** 10.1186/1471-2458-6-202

**Published:** 2006-08-04

**Authors:** Helen Wormald, Heidi Waters, Mike Sleap, Lee Ingle

**Affiliations:** 1 Specialist Health Promotion Service, Hull and East Riding Primary Care Trusts, Victoria House, Park Street, Kingston-upon-Hull, HU2 8TD, UK; 2 Department of Sport, Health and Exercise Science, The University of Hull, Cottingham Road, Kingston-upon-Hull, HU6 7RX, UK; 3 Department of Academic Cardiology, The University of Hull, Castle Hill Hospital, Castle Road, Cottingham, Kingston-upon-Hull, HU16 5JQ, UK

## Abstract

**Background:**

The health benefits of an active lifestyle have been extensively documented and generally accepted. In the UK, declining physical activity levels are a major contributing factor to a number of public health concerns such as obesity and coronary heart disease. Clearly, there is an urgent need to support people in developing sustainable active lifestyles. In 2003, a new lifestyle-based physical activity service called *Active Lifestyles *(AL) was set up in Kingston-upon-Hull to help local residents to become more active and develop healthier lifestyles. The service targeted the most deprived communities in the city. The aim of the study was to explore participants' perceptions of the operation and effectiveness of the AL service.

**Methods:**

Five focus groups were conducted in community centres and offices in the health promotion service in Kingston-upon-Hull. Sixteen white adult males (*n *= 5) and females (*n *= 11) participated in the study. Ages ranged from 15–73 years (mean age = 53 years). Data were analysed using a content analysis technique based on the 'framework' approach.

**Results:**

Three broad themes emerged from the focus groups; the referral process; operational aspects of the AL service; and perceived benefits of the service. Overall, participants were extremely positive about the AL service. Many reported increased activity levels, modified eating habits, and enhanced awareness and education regarding healthier living. Most participants reported that local awareness of the AL service was low and greater promotion was required so more people could benefit. The success of the service was highly dependent upon the qualities and approach of the AL advisor.

**Conclusion:**

The service appears to have filled a gap in service provision since it offered support to the most sedentary, older, unfit and overweight individuals, many of whom live in the most deprived parts of Kingston-upon-Hull. Traditional exercise referral schemes that focus solely on facility-based exercise should be broadened to encompass everyday lifestyle activity, where referral to a gym or exercise facility is just one of a number of physical activity options.

## Background

The health benefits of a physically active lifestyle have been extensively documented and generally accepted [1]. The majority of the UK adult population do not engage in sufficient physical activity to reap these health benefits [2], and despite a variety of promotional efforts, there are few examples of public health initiatives that successfully achieve long-term increases in physical activity [3]. Since declining physical activity levels have been strongly associated with a number of public health concerns such as obesity and coronary heart disease, there is an urgent need to support people in developing sustainable active lifestyles.

One type of physical activity intervention that has grown in popularity is the exercise referral (ER) scheme [4], which usually involves a health professional referring a patient to a short-term programme of exercise in a leisure centre or gym. These schemes can be beneficial for some people in terms of increasing physical activity levels and improving health and well-being [3,4], and while patients themselves view ER schemes positively and report increased activity and improved health and well-being [5], they are not for everyone [4]. For the most inactive, unfit, older or overweight individuals, exercising in a gym might not be the most appropriate, preferred or safe option. Furthermore, many people drop out of facility-based exercise programmes within six months [6,7]. Recent evidence indicates that a much broader, lifestyle approach to promoting physical activity would be more effective, with greater emphasis on being more active during everyday life including activities such as walking, gardening and taking the stairs, and with less dependence on leisure-based facilities [3]. Hillsdon and colleagues [3] also indicated that interventions needed to be based on individual behaviour change theory, using strategies such as goal-setting and self-monitoring, tailored to individual needs. They further suggested that regular and ongoing contact with a physical activity advisor was important, as was the promotion of moderate intensity physical activity.

In light of this evidence, a new lifestyle-based physical activity referral service was set up in Kingston-Upon-Hull, UK, in 2003 to help local residents to become more active and develop healthier lifestyles. The *Active Lifestyles *(AL) service involves health professionals referring patients to an AL advisor for individual advice and support. Referral criteria includes patients aged over 12 years with a sedentary lifestyle and/or a range of mild to moderate physical or mental health problems such as being overweight, obese, or suffering from hypertension, anxiety or depression. The service is specifically targeted at those needing motivational support for behaviour change. The AL service complements the existing, more traditional ER scheme in Kingston-upon-Hull, which provides a short-term (10-12-week) programme of exercise in a gym or class setting.

**Figure 1:**
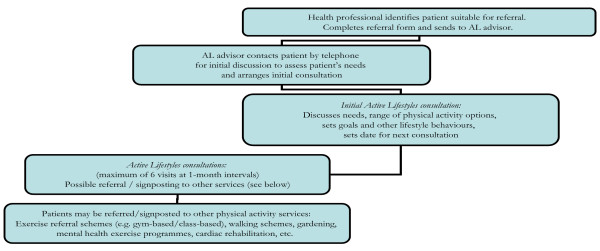
Active Lifestyle Service – Typical Referral Pathway.

Figure 1 illustrates the typical referral pathway for patients in the service. Completed referral forms are sent by the health professional to the AL advisor using the internal National Health Service (NHS) mailing system. An information leaflet, which is given to the patient by the health professional, introduces the service and clarifies what to expect at the consultation. Individual consultations are held in GP surgeries, community centres and schools. The first consultation lasts approximately one hour, and subsequent consultations are generally shorter. The AL advisor provides motivational support to help the patient become more active through behaviour change strategies and individual lifestyle changes such as more walking or increased activity around the house. Patients are provided with a goal-setting sheet on which they record their activity goals, potential barriers to success and strategies for overcoming them. Patients are asked to sign these sheets to show their commitment to the goals and aid motivation. Where appropriate, the AL advisor can also refer patients to a range of organised activities including walking groups, green gyms and ER class or gym schemes. Continuing support is offered to the patient by the advisor in the form of up to six progress consultations at monthly intervals, and optional telephone advice. In addition, patients are provided with physical activity diaries and, where appropriate, food diaries to help them to monitor progress. The service is suitable for those who are lacking in confidence or motivation, or who simply do not know where or how to start changing their lifestyle. Following each consultation the advisor completes a 'patient progress sheet' containing physical measurements (e.g. blood pressure, bodyweight) and a brief written report about the patient's progress. This information is useful for tracking patients' progress over time. Progress sheets are sent to the referring health professional after each consultation, and a copy is also kept by the AL advisor.

Kingston-upon-Hull is ranked as the ninth most deprived local authority region in the UK, with high levels of unemployment, low educational attainment and low home ownership [11]. The wards where the AL service was initially targeted are amongst the one percent most deprived wards in the UK, which is in accordance with the current focus of the National Health Service to reduce health inequalities. Research has indicated that physical activity levels are lowest among those with low levels of education and people in low-income households [8]. Hillsdon and co-workers [3] reported that there is no evidence regarding the effectiveness of physical activity interventions among these social groups. Indeed, Taylor [9] expressed the need for investigation of the effectiveness of ER schemes 'in settings where high levels of social exclusion and poverty exist' (p.173). In order to engender long-term lifestyle changes in these groups, it is thought that mediators such as improved self-efficacy, enjoyment and enhanced social support need to be addressed [10].

Funding to establish the AL service was initially provided by the Kingston-Upon-Hull Neighbourhood Renewal Strategy, and later the Eastern and West Hull Primary Care Trusts. In the two-year period since the AL service commenced, one advisor has supported over 180 people. The advisor was recruited based on the requirements of the post. She possessed a relevant degree, certificates in GP exercise referral and cardiac rehabilitation, and had experience of working in a gym setting with referred patients. Many of the service users shared common characteristics including multiple physical and mental health conditions, obesity and overweight, high levels of unemployment, and personal issues such as suicidal thoughts, alcoholism and domestic violence. As the AL service was new, it was essential to monitor and evaluate its effectiveness. While a range of quantitative data were collected including demographics and physiological and behavioural changes, it was felt that it would also be useful to gain feedback from patients themselves, to ascertain their opinions of the service. The aim of the study, therefore, was to explore participants' perceptions of the operation and effectiveness of the AL service in Kingston-upon-Hull.

## Methods

### Setting

Five focus groups were conducted in community centres and offices of the health promotion service in the city of Kingston-upon-Hull, UK.

### Participants

Sixteen white adult males (*n *= 5) and females (*n *= 11) participated in the study. Ages ranged from 15–73 years (mean age = 53 years). All had attended at least one consultation with the AL advisor.

### Procedures

The Hull and East Riding Research Ethics Committee granted approval for the study. By selecting names at random intervals, a sample of patients was selected from a list of service users. All selected patients had attended at least one consultation with the AL advisor. Letters, information sheets and informed consent forms were sent to each patient. The letter outlined the purpose of the study and requested that they read the information sheet and sign and return the informed consent form. The information sheet provided study details, including timescales and expectations of each participant. The informed consent form required participants to indicate whether of not they were willing to participate, and to provide a name, signature and date. A range of dates was offered to the prospective participants for attending focus groups. A total of 110 letters were sent out, and 29 informed consent forms were returned, indicating a response rate of ~26%. Of the 29 people who responded 19 agreed to participate, and their GPs were informed of their intended participation. Of these, 16 attended on the days.

The method of data collection utilised was the focus group, which has been recommended as a suitable tool for exploring a range of opinions in health research [12,13]. Focus group topics were developed, revised and subsequently agreed by members of the AL research team. Focus groups lasted between 45–65 minutes, and between one to seven participants were involved in each group. Participants were given £10 gift vouchers and were promised details of study findings. Two experienced facilitators conducted the focus groups. The purpose of the focus groups was explained to participants, and it was emphasised that all data would be anonymous. Discussions were audio taped with permission from participants and transcribed verbatim.

### Data analysis

Data were analysed using a content analysis technique based on the 'framework' approach [13]. A researcher read the transcripts several times in order to become familiar with the material and identify emergent themes. A second researcher repeated this process, and, following discussion, a thematic framework was devised. The relevant section of text was cut and pasted on flipchart paper under the relevant theme. This approach has been previously criticised as it involves the removal of text from its original context [12,13] and therefore, each section of text was referenced so it could be traced back to its original source.

## Results and discussion

Analysis of the focus group data revealed the following broad themes:

*1. The referral process*.

*2. Operational aspects of the Active Lifestyles service*.

*3. Perceived benefits of the service*.

The broad themes were subsequently split into sub-categories, which are discussed in detail below. Due to the qualitative nature of this study, the aim of the discussion will be to present a range of opinions rather than quantifying them. However, where possible an indication will be given as to whether an opinion was held by, for example, the majority of participants, or only one. Where quotations have been used to illustrate a point, no names have been provided, only gender and age. The name of the staff (AL advisor) has been changed.

### 1. The referral process

#### Reason for referral

As with other schemes [14] participants had been referred to the AL service for a wide variety of physical and/or mental health problems. Some of these included arthritis, hypertension, depression, heart disease, Crohn's disease, asthma, diabetes, bowel meningitis, and back pain. When asked why they had been referred, many participants gave long lists of health and other problems:

"I was riddled with arthritis, heart attacks and Crohn's disease."

(Male, 64 years)

Participants mentioned weight loss or weight management frequently as a reason for referral, echoing the findings of Hammond and colleagues [15]. Even if not the main reason for referral, it was certainly an issue for almost all participants. A few said that they had put on weight after stopping smoking and had then been signposted from the smoking cessation service to the AL service for help with physical activity and lifestyle change:

" That's really why I went because I'd stopped smoking and I'd put on all this weight and I thought I'm going to change my life and do something ... do a bit of exercise."

(Female, 73 years)

Certain life events or achievements such as stopping smoking, retirement or reaching a milestone birthday appear to have spurred participants to seek help and change their lifestyle:

"I was 40 this year which got me talking to my GP because I didn't want to just get bigger and bigger, I just wanted to do something about it."

(Male, 40 years)

It became apparent during the discussions that, while many participants had been referred for a particular reason, they often had more complex needs. As well as having multiple health problems, many other personal and social issues were raised. One male participant, for example, was referred to lose weight and reduce blood pressure, but throughout consultations with the AL advisor he raised issues such as his own depression, suicidal thoughts, unemployment and illnesses of close family members. This participant had gained much from the AL service, including a more positive attitude to life. It was evident that participants gained very different benefits from the service and these issues will be discussed further in the '*Perceived benefits of the service*' section.

#### Source of referral

A GP or practice nurse had referred most participants to the scheme, with the remainder being referred by a dietician. Some participants had approached their GP or nurse themselves for a referral after picking up an AL leaflet from a surgery. One participant commented that she had struggled to be referred to the service as the GP had refused to sign the referral form:

"She (the dietician) told us about Active Lifestyles and then we went to our local nurse and she referred us here, but our doctor wouldn't sign it for ages because he said there was no point."

(Female,15 years)

#### Access to the scheme

General public awareness of the scheme appeared to be low. Many participants noted that they had not heard about the service before the health professional had mentioned it, while others were aware that it existed but did not know the details:

"I had heard about it ... a lot of people were talking about it but nobody seemed to know anything about it, like exactly what you did. I didn't know what it was about or what it did until my doctor referred me."

(Male, 50 years)

Participants' opinions of the health professionals' knowledge of the service differed; a few participants felt their health professional had been a strong advocate of the service, and had seemed reasonably well informed about it:

"I had a long chat with my GP and he said there was this new scheme starting and do you want to be referred. I didn't know anything about it until the doctor told me about it ... he was really for it. Yes, he explained everything."

(Male, 40 years)

The majority of participants, however, felt that although most of the health professionals were aware of the service, they did not appear to be well informed of what it entailed and only provided brief, if any, information about the service. Furthermore, a few participants were disappointed that their health professional had not asked them of their progress with the service, and had shown little interest:

"I have been to see the GP for a few times after I have been to Active Lifestyles and not once has she asked how I have got on. So what is the point, the doctors are not interested – they don't want to know."

(Female, 73 years)

This reflects Taylor's [16] conclusions from a number of studies that found there was a lack of awareness among some GPs to promote physical activity.

Many of the participants had not been given an information leaflet upon referral, so had no idea what to expect from the initial meeting with the advisor. This led to anxiety among participants who went to consultations not knowing what they would be asked to do, or if they would be doing any physical activity. Many also mentioned how they had expected a very different approach to the one they received – they thought the advisor would take a more forceful and authoritative approach:

"I was a bit nervous about it thinking oh she's going to say right go to the gym, do this, this is what you need to do, but when I met her it was none of that."

(Female, 24 years)

"I didn't expect it to be like it was. I expected someone who was going to say right you're going to do this, you've got to do that, but she's not like that at all, she's really nice."

(Female, 51 years)

Some of these preconceptions appear to be the result of past experiences, where other health professionals have taken a more authoritative and stricter approach:

"When I met my dietician she said right you've got to stop chocolate, you've got to stop crisps, you've got to stop that, and I just thought she ('Sarah') was going to be like if you don't go to the gym what do you expect?"

(Female, 24 years)

Throughout discussions the most common suggestion for improving the service was that it needed to be promoted more, a recommendation also made by Hammond and colleagues [15]. Almost all participants felt that awareness of the service was low, and that a lot of people who could benefit from it did not know about it. Suggestions of how to promote the service included displaying posters and leaflets in GP surgeries, hospitals, libraries, supermarkets, community and shopping centres, post offices, shop windows and schools. Media sources such as local radio or the BBC bus were suggested. Participants also felt that older, unfit or unhealthy people should be targeted, as these people might not realise that the service was suitable for them. In addition, it was acknowledged that if awareness among the health professionals themselves were improved then more people would be offered the service.

### 2. Operational aspects of the AL service

#### Service provision

The AL service currently offers a maximum of 6 consultations per patient, which are generally held at monthly intervals. Very few of the participants had seen the AL advisor more than 4 times, with many having seen her only 2 or 3 times and then feeling confident of continuing on their own. When asked their views on consultations being held monthly, the majority of participants felt that this was about right – that meeting any sooner would not allow enough time to make sufficient changes, and that a longer interval would mean that they would lose motivation:

"Yes I liked it monthly because I felt as though you wasn't being watched and that you got chance to do everything you wanted. It wasn't a rushed thing thinking right I've got to get it done because I've got to see her next week, but I felt as though you saw more improvement to report to her."

(Female, 24 years)

This concurs with the findings of Hillsdon and Thorogood [17] that regular follow-up increases the likelihood of sustained behaviour change.

One interesting finding from the discussion was that many of the participants felt that the service should be ongoing, and not limited to a certain number of consultations or a specific time period. While very few had needed six consultations, they were not in favour of the service being limited and being unable to see the AL advisor for 'check-ups':

"But after the sessions I wouldn't like to think that it is finished full stop, and that you're in the filing cabinet. I would like to think that you could go at least twice a year ... as you would to a doctor for a check-up. To go back to 'Sarah' just to see whether you had lapsed in anything, if you have forgotten anything, or if there is anything new on the market so to speak."

(Male, 67 years)

Between appointments the AL advisor offered telephone support as a way of helping participants stay motivated. Feedback during discussions suggested that while most participants found telephone support a very helpful and motivating aspect of the service, others did not and would not have been happy having someone *"ringing me up and saying are you doing this? Are you doing that? " *It appears that optional telephone support is appropriate and should remain as it is.

While not all participants were asked about this issue, as it only arose during one discussion group, those who were asked felt very strongly that the service and support should be ongoing. This issue should be explored and the implications of offering the service on a continuing basis should be identified. While on the one hand the participants clearly felt that this was important, and that without it they might fall back into old habits, there is also a need to encourage people to take responsibility for themselves and their health, and not become too dependent on health staff or services. This issue has been previously raised by Hunt and Hillsdon [18]. Furthermore, if participants were able to continue seeing the AL advisor it would increase demand and time pressure on an already stretched service.

The dependency that a lot of participants clearly have for health services and staff was apparent during discussions regarding other aspects of the service. Some participants suggested that having their measurements taken by the AL advisor at consultation, such as blood pressure and body weight, helped them to stay motivated, and that without this they would not have stuck to their goals:

"It makes you feel more positive about it, when you've got somebody there. If you could see someone regularly you know you're going on the scales, because if you try to diet for yourself you just cheat and you're only cheating yourself."

(Female, 51 years)

Most participants found the tools such as the physical activity diaries extremely useful in helping them to stay motivated. The physical activity diary acted as a goal-planner, enabling participants to keep a record of their goals and to be able to tick them off once they had achieved them:

"They (diaries) really help me because it's like I've done that today and it did sort of remind me that well I need another walk to do ... I do 20 minutes walk 5 times a week so I used to think, right I've done 4 I'm alright, I'll go out tonight. I just stick them on my fridge."

(Female, 24 years)

"You put your goals down, like how many times you walked the dog in a week, how many you're aiming for, and then you tick them or write how much time you have done. Yes, I did (find it useful)."

(Female, 15 years)

This illustrates the potential of goal setting and self-monitoring in the maintenance of new physical activity behaviours, as has been previously highlighted by Biddle and Mutrie [19]. Only one participant said that although she had found the diary useful initially, she had not used it after a while. The healthy eating diary also appeared to help participants to be more aware of what they were eating and drinking, and thus help them to modify their eating behaviours.

Although the AL service offers a number of venues in which to see the advisor, a few participants said that they would have preferred a greater range of options. Due to the fact that many participants relied on public transport some had found it difficult to access the service.

Some participants were aware that the AL advisor was very stretched in trying to deal with the high demand for the service, and thought that the service should be expanded and more AL advisors recruited. Despite this awareness of how busy the AL advisor was, one of the most appreciated aspects of the service was that participants felt that she had time for them, compared to their experiences of other health services, which they felt were too rushed:

"It's just that she doesn't really have time for you ... my dietician. When you go in you sit there and she goes right let's get you weighed, then she'll say right have you been following your diet, and if you say yes then she goes ok then see you in 4 to 6 weeks, and that's it ... it's more like seeing the doctor."

(Female, 24 years)

A key aspect of the AL service is that it acts as an information source and signposting service, and many of the participants had been referred from the AL service to other physical activity services. This did not always happen initially, and many participants had been supported to increase their activity levels gradually, perhaps by doing more walking and home-based activities. Later, they had been referred to other services. A few participants had merely wanted information on physical activity services such as Tai Chi classes or walking groups. Many others had been referred to the ER scheme by the AL advisor – either to the exercise classes or the gym, and some had been to both.

Overall, participants were extremely happy with the AL service, and many felt that there was little that could be done to improve it, and their main concern was that it did not cease to exist, for example due to funding cuts. Interestingly, one participant was pleased that AL was an NHS-run service:

"...knowing that the NHS was behind the scheme....it was reassuring to know that the NHS was trying to do something about it – rather than just looking after sick people they're actually trying to alter lifestyles."

(Male, 40 years)

#### The Active Lifestyles advisor

A crucial finding from this study was that the most important element of the AL service appeared to be the AL advisor – the personality and approach of the advisor is likely to determine the success or failure of the service. This reiterates the value of a counselling approach involving cognitive-behavioural strategies that can maximise adherence to higher physical activity levels [20].

Overall, feedback from participants about the AL advisor was extremely positive – they were full of praise for her approach. They noted how she was caring, supportive, sincere, knowledgeable, and a good communicator and listener. Furthermore, she was genuinely interested in them as people, and provided a very personal service. This made a big impression on many of the participants, helping to boost their self-confidence and giving them a feeling that someone actually cared about them and had time to listen:

"Actually interested in you as a person, and it didn't seem like it was just a job – 'Sarah' really does care. There's an awful difference when you're doing something and just going through the motions, like 'do this' and 'do that'. She's not like that at all."

(Female, 51 years)

"I think she's a really caring person, she really wanted to help."

(Female, 37 years)

Participants appreciated the advisor's non-judgemental manner, an approach strongly encouraged by Stott and Pill [21]. Instead, she had worked with them and helped them to make changes in a very supportive and empowering way. Rather than telling them what they should do she helped them to discover answers themselves:

"Oh yes, 'Sarah' didn't stand there with a big stick ... 'you will do this', 'you will do that', it was 'look, how do you feel about doing it?' 'how do you feel about this?' 'how do you feel about that?"

(Male, 50 years)

"She actually knew what she was talking about, she was very supportive and if you asked her a question she could answer it for you."

(Male, 40 years)

The majority of participants had greatly appreciated the gentler approach of helping them to start very slowly and build up gradually:

"She said if you're poorly and you haven't done it that week, don't feel bad about it just do it from next week. She showed me things to do even if I'm just laid up in bed, in hospital ... try and do little things."

(Female, 24 years)

By encouraging participants to progress gradually and not push themselves too much at first, the advisor also helped to dispel some of the myths about physical activity. She showed them that, as advised by Blair[22], a low to moderate level of activity can be very beneficial and that it is not necessary to go to the gym or engage in vigorous exercise to gain health benefits:

"I thought well if you don't go to the gym it's not worth doing anything, but she proved that it's not like that."

(Female, 24 years)

Other qualities that participants appreciated in the advisor were that she was knowledgeable about the subject, and if she ever did not know something she would find out for them. They also felt that she was a good role model and was reliable, for example in returning their phone calls.

The AL advisor appears to have achieved an appropriate balance between providing a professional service but also being down-to-earth, caring, sincere and likeable at the same time:

"It is more like a friendship relationship with 'Sarah' rather than a health person, and you don't feel as though she is instructing you."

(Female, 39 years)

### 3. Perceived benefits of the service

#### Improving physical activity levels

Most participants felt that they were more active as a result of the AL service. Many had been helped to make small but significant, gradual changes to their activity level. Although this paper does not offer statistical evidence, the perceptions of this sample support the conclusions of others [23], that referral services can make at least a small positive improvement in physical activity levels. The advisor had helped them to think differently, and to be creative in finding ways of building activity into their lifestyle. One young female participant, for example, explained how she was more aware of being active at school, so instead of standing around in the corridor during break time, she walked around the playground. Other participants had fitted activity into their own normal routines:

"I'm doing exercises as well as housework. It's not much but just a little bit of extra walking – instead of taking a basket into the garden to hang the washing up, just go in and out for each item. It's just a little bit more. I've got a pedometer as well – she encouraged me to get a pedometer, and when you see that going up it makes you do more. I'm a lot more active than I was and I feel a lot better."

(Female, 51 years)

"I've started walking to the shops, where I took the car in the past. The lady I look after lives on Hessle Road so I walk down Hessle Road with her, I push her in her wheel chair. She's got two dogs and I take them out for her on a night and we go for an hour."

(Male, 40 years)

Participants who had been relatively active at the start, or had built up their activity or confidence, had then progressed to other activities such as exercise classes, gym visits or swimming:

"I was thinking yesterday when I was in the gym ... when I first started 'Sarah' wanted me to do ... at least thirty minutes a week, and now I'm doing god knows how many hours a week. I thought good grief, how far have I come since that first meeting with 'Sarah'. I'm doing exercise and I'm actually enjoying it!"

(Male, 40 years)

"It is really good. I am more active now than I was three or four years ago. I just go on walks not take my car. Take the dog out four or five times a day."

(Male, 64 years)

A lot of participants commented that they had influenced their family or friends through encouraging them to be more active or healthy, and many of them took part in activities with family members or friends:

"Yes I take my granddaughter swimming which I didn't do before. I do a lot of things now that I didn't do before, and my wife does as well."

(Male, 67 years)

While a few participants mentioned their fears of slipping back into old habits, particularly when the weather got colder, the majority appear to have changed not only their physical activity behaviour but also their outlook and way of thinking. Many felt that they would be able to continue with the changes for the long term. This new outlook had come as a result of time spent with the advisor. Many participants noted how they had learnt such a lot about physical activity and healthy lifestyles, thus changing their preconceived ideas:

"...like the treadmill, I thought you had to be running on them, but I'm just walking and he (the gym instructor) said look and it shows you how many calories you're burning off and things like that, and I didn't realise, I just thought treadmill – you've got to run. ... I didn't know until someone showed me and that's what this has done for me – it has changed me as a person, and it has changed my lifestyle, my husband's and my son's ... I feel so thick because I didn't know all this went on."

(Female, 24 years)

#### Improved health and fitness

As a result of increased physical activity and other lifestyle changes, participants had noticed a range of benefits including improved health and fitness, feeling less stiff and having greater mobility, finding exercises easier and having stronger, more toned muscles. Many participants mentioned weight loss, or having been able to maintain their weight:

"It's great today, I went for a pair of trousers that were really tight on me and I can get into them easily now and that's such a boost when you do that."

(Male, 40 years)

#### Healthier lifestyles

Although the AL service was designed as a physical activity service, it provides support for other lifestyle behaviours and in many other ways. For example, through discussions with the advisor, many participants had been asked about issues such as food, smoking, and stress, and had received information or advice relating to their needs. Many participants had discussed their eating and drinking habits, and some had completed food diaries to help raise awareness of where they could make changes. As a result of the information that the advisor provided, a lot of participants had changed their eating habits and had started buying different foods:

"Before you see I used to just pile it on my plate – I would eat as much as I wanted to eat and I wouldn't really be so conscious about fruit and veg. ... Now, yes I do eat the food I like and I have gone more onto liking fruit and veg."

(Female, 24 years)

"I eat more healthily now than I probably have done for ages."

(Male, 40 years)

#### Self-confidence

One of the most apparent benefits was the development of self-confidence and having a more positive outlook:

"I am better in myself. I feel more confident. I'm not so nervous."

(Female, 24 years)

"*She's given me a lot more confidence. Just talking to her after the first session I went home with a more positive attitude."*

(Female, 51 years)

The development of confidence or self-efficacy is well accepted as a central feature in the adoption and maintenance of physical activity behaviour [19]. For one participant, the AL service had empowered her to change her attitude and feel happier in herself and accept herself as she was:

"She ('Sarah') hasn't made me a selfish person, but my marriage has changed because I was doing everything for my husband, trying to get thin for him. But I thought no, I'm not bothered if I'm thin ... I want to do this for me, and he's seen a change in my attitude, but probably on his side he thinks no this isn't for the better, but for me it is."

(Female, 24 years)

#### Other benefits

For a few, they had merely wanted information on activities that were available, and the AL service was able to put them in touch with other services or opportunities for exercise where they could meet people and make friends. One participant was new to the area and wanted to meet new people, and another had recently retired and wanted something to do. Some noted how the service 'filled a gap' in their lives, and that attending classes 'breaks up the day' and gives something to look forward to:

"I found that she ('Sarah') was a link to me doing what I wanted to do which was this keep fit (exercise referral) class, getting out, meeting people. Once you're retired you do find it very hard, it's very boring ... I think you need to get out to meet people ... the keep fit is a social occasion."

(Female, 71 years)

Participants gained very different things from the service. Many of them came to the service with a whole range of health, emotional and social problems, and thus have benefited in a number of ways. Some have made small lifestyle and behaviour changes to increase their activity, while others have gone on to join exercise classes or gyms, and perhaps made friends. Some felt fitter and healthier, more mobile and better able to cope with the activities of daily living. Some considered that they had a much more positive attitude to life, and felt more confident in everyday life and relationships. Some had been educated about health and activity in a way that had changed their lives. A few just needed someone to talk to, and physical activity gave them something positive to focus on:

"If I am feeling a bit negative I could go and see 'Sarah' and she is going to say well look, forget about the negative side, look at the positive side. You know like I feel like saying sod it and jumping out of the window I have been that depressed at times but 'Sarah' said no don't do that. Sit down and think about it and look at what you need to do."

(Male, 50 years)

The many benefits of the service reflect the personalised approach taken and the varied needs of the participants. While this is a valuable aspect of the service, there is a need to consider what the AL service can and cannot achieve. AL is not a counselling service, and the staff are not trained to provide counselling. However, it is important that the service maintains links with other supportive services and professionals, so that patients can be referred if necessary. This study has also highlighted the need for services that provide someone to listen, support and advise, whether this is through AL or a different setting. Many participants in this study were not necessarily in need of a psychologist or counsellor, but greatly benefited from someone who had time to listen to them as well as supporting them with lifestyle changes:

"Until I talked to my GP I felt there was no help out there, nobody seemed to be interested – just go away and try and diet. But 'Sarah' has encouraged me in a big way, she's got it through to me that it's not just dieting, it's healthy living – try and change your lifestyle by your eating habits and try to be more active and get out there and do it for yourself."

(Male, 40 years)

"She's helped me a lot. She has inspired me to go on to physical activity that I didn't think I would ever achieve really, again, but she's been a great benefit."

(Male, 64 years)

## Conclusion

This study aimed to provide preliminary findings of participants' perceptions of the operation and effectiveness of the new AL service in Kingston-upon-Hull. It is felt that many of the issues highlighted in this article are generic issues that could be related to similar schemes around the UK. It is hoped that the recommendations in Table 1 will provide useful, practical ideas for improving the effectiveness of similar schemes around the UK.

Overall, participants were extremely positive about the AL service and appear to have benefited in a number of ways. Many had increased their physical activity levels, changed their eating habits and had developed more positive attitudes and fulfilling lives. Many had become more educated regarding healthier living, and had lost their negative preconceptions of exercise.

Most participants reported that awareness of the AL service was low and greater promotion was required so more people could access and benefit from it. One of the most important findings from the study was that the scheme's success appeared to be dependent upon the qualities and approach of the AL advisor. It is essential that the advisor is empathetic, non-judgemental, sincere and knowledgeable. The approach needs to be empowering and supportive, in order to build confidence and self-belief.

The AL service appears to have filled a gap in service provision and provided a much-needed service to those who could benefit from it the most. It provided support to sedentary, older, unfit and overweight individuals, many of who lived in the most deprived parts of Kingston-upon-Hull. Without this service it seems that many people would have had nowhere else to turn to for help and support with making initial, small steps towards healthier, more active and fulfilling lives. Many would probably not have accessed the more traditional ER service, at least not initially and without support. The AL service is inclusive as it provides free support to individuals with a range of needs and circumstances, and can provide information on, and access to, a range of physical activity options to meet a wide range of needs and individual circumstances.

Traditional ER schemes that focus on facility-based exercise should be broadened to encompass everyday lifestyle activity, where referral to a gym or exercise facility is just one of a number of physical activity options. Services could also provide support for changing other lifestyle behaviours, particularly healthier eating habits for those wishing to lose weight.

## Abbreviations

AL – active lifestyles;

ER – exercise referral;

## Competing interests

The author(s) declare that they have no competing interests.

## Authors' contributions

HWo was responsible for the overall planning and design of the research project, assisting with conducting the focus groups, as well as analysis, interpretation and writing up of the data. HWa was the AL advisor who delivered the service to patients on a day-to-day basis. MS and LI were responsible for drafting and revising the manuscript for important intellectual content.

## Pre-publication history

The pre-publication history for this paper can be accessed here:



## Figures and Tables

**Table 1:** Summary of Recommendations

• Ensure a sufficient number of staff (i.e. physical activity advisors) are employed to meet service demands, and that staff have enough time to support patients effectively.
• Ensure that physical activity advisors have the appropriate skills and qualities (e.g. empathy, reliability, sincerity, knowledge) and that the approach taken is empowering and supportive.
• Services should be sufficiently promoted e.g. via posters and leaflets in GP surgeries, hospitals, libraries, and use of local media.
• Marketing should target unfit, older, overweight individuals.
• Ensure health professionals (e.g. GPs, practice nurses, dieticians) have sufficient information and awareness about the service.
• Upon referring a patient, health professionals should provide the patient with information (e.g. a leaflet) about the service so they know what to expect.
• Ensure that an appropriate number of accessible venues are available for consultations.
• Consultations with advisors should be at monthly intervals.
• Telephone support should be an optional aspect of the service.
• Patients should be encouraged to start slowly and build up activity and confidence gradually.
• For patients wanting to lose weight, provide support for changing eating as well as activity behaviours.
• Provide physical activity and food diaries to help patients to monitor progress.
• Explore the implications of providing a continuing service e.g. optional bi-annual 'check-ups' with advisors.
• Ensure the service links with other supportive services and staff e.g. smoking cessation, dieticians, counselling.
• Seek to secure ongoing funding for exercise referral services.
